# A novel quantitative real-time PCR diagnostic assay for fecal and nasal swab detection of an otariid lungworm, *Parafilaroides decorus*

**DOI:** 10.1016/j.ijppaw.2020.04.012

**Published:** 2020-05-18

**Authors:** Kalani M. Williams, M.K. Fessler, R.A. Bloomfield, William D. Sandke, Clara R. Malekshahi, Caroline D. Keroack, Pádraig J. Duignan, Samantha D. Torquato, Steven A. Williams

**Affiliations:** a Smith College, Department of Biological Sciences, Northampton, MA, 01063, USA; b The Marine Mammal Center, Sausalito, CA, 94965, USA; c University of Massachusetts, Molecular and Cellular Biology Program, Amherst, MA, 01003, USA

**Keywords:** Pinniped, Marine mammals, Nematodes, Lungworm, *Parafilaroides decorus*, Quantitative PCR, Molecular diagnostics

## Abstract

*Parafilaroides decorus,* also known as sea lion lungworm, is a metastrongyloid nematode that infects otariid hosts, such as the charismatic California sea lion, *Zalophus californianus. P. decorus* causes bronchointerstitial pneumonia, respiratory distress, reduced ability to swim, dive and hunt and as a result, increased mortality particularly in young animals. Respiratory disease is a leading cause of stranding and admission to rehabilitation centers on the Pacific coast. Low-coverage genomic sequencing of four *P. decorus* individuals analyzed through Galaxy's RepeatExplorer identified a novel repeat DNA family we employed to design a sensitive quantitative PCR (qPCR) assay for diagnosing infections from fecal or sputum samples. The assay detects as little as 10 fg of *P. decorus* DNA and a linear regression model developed using a standard curve can be used to estimate the concentration of *P. decorus* DNA in a sample, ± 0.015 ng. This knowledge can be leveraged to estimate the level of parasite burden, which can be used to design improved treatments for animals in rehabilitation. Improved treatment of infections will aid in more animals being successfully released back into the wild.

## Highlights

• Developed qPCR assay for *P. decorus*.
• Sensitive to 10 fg.
• Applicable for feces or sputum.

## Graphical abstract

**Figure:**
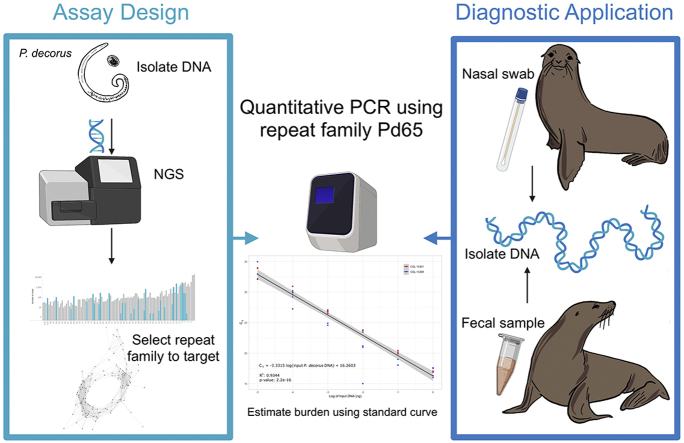

## 1 Introduction

To the detriment of much marine life, global warming is predicted to increase the range, prevalence, and virulence of aquatic parasites (Harvell et al., 2002). Accurate identification of these parasites can allow for better assessment and treatment of pathogenic threats to wild marine mammals and treat infections of those being cared for in rehabilitation facilities. Our goal is to create sensitive, species-specific assays for diagnosis of pre-patent infection in California sea lion (*Zalophus californianus*) patients who present with respiratory disease and to assist clinicians in monitoring the efficacy of anthelminthic treatment (Field et al., 2018). Current diagnostic practice is to use a Baermann test to detect larvae in feces, but the test can only detect a patent infection and it lacks sensitivity and specificity and is not quantitative (Pilotte et al., 2016, 2019; Easton et al., 2017). While PCR has been repeatedly demonstrated as superior in both sensitivity and specificity, the use of traditional ribosomal or mitochondrial DNA targets can also be insufficiently sensitive in detecting trace amounts of DNA from parasite eggs or larvae in feces (Pilotte et al., 2019). By using a repeat-based quantitative PCR (qPCR) assay with a higher number of genome targets, assays are not only more sensitive but allow for the estimation of parasite burden (Pilotte et al., 2019). In nematodes, these non-coding repeats evolve quickly enough that they tend to be species-specific (Pilotte et al., 2016). *Parafilaroides decorus,* a common lungworm of otariids, is a metastrongyloid nematode. The adults are found in the bronchioles and alveolar parenchyma of sea lions of all ages but are particularly prevalent in pups and juveniles (Greig et al., 2005). In most animals *P. decorus* causes minimal inflammation and only mild clinical signs such as coughing as larvae are expelled from the airway (Measures, 2018). However, heavy parasite burdens in young animals are characterized by bronchitis, bronchiolitis and interstitial granulomatous pneumonia, which reduce the ability to swim, dive, and hunt, and result in increased mortality in these young animals. Bronchioles are often obstructed by nematodes causing inflammatory exudate and thick mucus. Infection is often complicated by opportunistic bacterial resulting in further pulmonary consolidation and often abscessation. The result can be severe dyspnea leading to stranding or death (Measures, 2018). In addition to pulmonary pathology, the life cycle of the parasite involves migration of larvae through the vascular system to the lung potentially causing fatal pulmonary endarteritis, thrombosis and infarction (Dailey, 1970; Seguel et al., 2018). While the primary host, the California sea lion (*Zalophus californianus*), has been rated ‘least concern’ on the IUCN Red List, close relatives of the California sea lion are also susceptible, including the Guadalupe fur seal, *Arctocephalus townsendi*, and the near threatened Steller sea lion, *Eumetopias jubatus* (Measures, 2018; Seguel et al., 2018).

*P. decorus* are ovoviviparous shedding first stage larvae into the lower airways where they can be observed in mucus droplets attached to the epithelium, and then moved by the mucus escalator to the pharynx, swallowed and shed in feces (Dailey, 1970; Measures, 2018). Either sputum or feces could be used for detection of larvae but the fecal Baermann test is the standard method (Measures, 2018). However, Baermann tests and microscopy are time-consuming, insensitive, and not species-specific (Pilotte et al., 2016). By using a species-specific qPCR assay, we can use the cycle at which a sample reaches exponential amplification, or the quantification cycle (C_q_ value), to estimate the amount of DNA from the targeted species in the sample. In this way, not only can the presence/absence of a particular parasite be determined from a sputum or fecal sample, but the infection intensity can also be estimated. To make such estimates, a predictive model must first be constructed. By isolating pure genomic DNA from a control *P. decorus* sample and using the assay to test a serial dilution of this DNA, we can obtain the data necessary to create a linear regression model of the relationship between input *P. decorus* DNA and the C_q_ value for this particular assay.

Parasitic burden can be inferred from the estimated DNA concentration in the feces or sputum. Armed with knowledge of both the species causing the infection and the burden of infection, marine mammal clinicians could choose the most appropriate therapeutic regimen for a particular patient and monitor the efficacy of antihelmintic treatment with sequential tests. These animals, if successfully treated, can then be released back into the wild, infection-free.

## 2 Materials and methods

### 2.1 Parasite, fecal, and sputum sample acquisition

*Parafilaroides decorus* and other nematode parasites were obtained from stranded, deceased otariids, while fecal and sputum samples were obtained from rehabilitating otariids collected by The Marine Mammal Center (TMMC, Sausalito, CA; Table 1). All host otariids were stranded on the Pacific coast of California. Each identification number represents the single host animal from which the nematode specimen was collected (Table 1). Negative control fecal and sputum samples were collected by the New England Aquarium (NEAQ, Boston, MA) and the Long Island Aquarium (LIA, Long Island, NY), respectively (Table 1). No parasites were obtained from live animals, and no live animals were harmed in the acquisition of samples for this study. All materials were obtained with a permit from the National Oceanic and Atmospheric Administration (NOAA) authorized under the regulations at 50 CFR 216.22(c)(5) and 216.37 of the Marine Mammal Protection Act, which states that marine mammal ‘parts’ can be transferred for scientific research purposes. All materials were collected in accordance with the NOAA regulations. All parasites were transferred from the rehabilitation facility to Smith College in 70–100% ethanol on ice. Fecal and sputum samples were transferred on ice without storage media. Upon acquisition, all samples were placed at −80 °C for long-term storage.

**Table 1:** Host data from whole nematode, fecal, and sputum samples. Note that ‘infection status’ refers specifically to *P. decorus* infections, not infections in general. Infection status was determined by a fecal Baermann test or morphological identification of the nematodes at necropsy. ^1^A whole nematode from each of these hosts was next-generation sequenced and used in the development of the assay. ^2^A nematode from this host was used as a positive control for the assay and was not used to develop the assay. *Aquarium animals constantly monitored by veterinarians and fed “sushi-grade” fish, should not be infected. Fecal and sputum samples from these animals were used as negative controls. TMMC = The Marine Mammal Center, LIA = Long Island Aquarium, NEAQ = New England Aquarium.

Host ID	Host species	Infection status	Whole nematode	Fecal sample	Sputum sample	Age class	Sex	Stranding date	Source
GFS-151	*Arctocephalus townsendi*	Infected	Yes^1^	Yes	No	Yearling	Male	07/06/2017	TMMC
CSL-13271	*Zalophus californianus*	Infected	Yes^1^	No	No	Juvenile	Male	10/30/2016	TMMC
CSL-13295	Zalophus californianus	Infected	Yes^1^	No	No	Subadult	Male	02/27/2017	TMMC
CSL-13301	*Zalophus californianus*	Infected	Yes^1^	No	No	Adult	Female	03/16/2017	TMMC
CSL-13302	*Zalophus californianus*	Infected	No	Yes	No	Adult	Male	03/17/2017	TMMC
CSL-13305	*Zalophus californianus*	No known infection	No	Yes	No	Adult	Female	03/22/2017	TMMC
CSL-13308	*Zalophus californianus*	Infected	Yes^2^	No	No	Subadult	Female	03/25/2017	TMMC
CSL-13534	*Zalophus californianus*	No known infection	No	Yes	No	Yearling	Female	10/22/2017	TMMC
CSL-13541	*Zalophus californianus*	Infected	No	Yes	No	Adult	Female	10/10/2017	TMMC
CSL-13564	*Zalophus californianus*	Infected	Yes	Yes	No	Subadult	Male	11/06/2017	TMMC
CSL-13591	*Zalophus californianus*	Infected	Yes	Yes	No	Adult	Male	12/03/2017	TMMC
CSL-14040	*Zalophus californianus*	Infected	No	Yes	Yes	Juvenile	Male	10/18/2018	TMMC
CSL-14066	*Zalophus californianus*	Infected	No	Yes	Yes	Adult	Female	11/04/2018	TMMC
CSL-14070	*Zalophus californianus*	Infected	No	Yes	Yes	Juvenile	Male	11/05/2018	TMMC
CSL-14073	*Zalophus californianus*	Infected	No	Yes	Yes	Subadult	Male	11/07/2018	TMMC
CSL-14075	*Zalophus californianus*	Infected	No	Yes	Yes	Yearling	Female	11/07/2018	TMMC
CSL-14083	*Zalophus californianus*	Infected	No	Yes	Yes	Juvenile	Male	11/10/2018	TMMC
CSL-14084	*Zalophus californianus*	Infected	No	Yes	Yes	Juvenile	Male	11/11/2018	TMMC
CSL-14089	*Zalophus californianus*	Infected	No	Yes	Yes	Yearling	Female	11/14/2018	TMMC
CSL-14107	*Zalophus californianus*	Infected	No	Yes	Yes	Yearling	Male	11/28/2018	TMMC
CSL-14117	*Zalophus californianus*	Infected	No	Yes	Yes	Juvenile	Male	12/05/2018	TMMC
CSL-14121	*Zalophus californianus*	Infected	No	Yes	Yes	Subadult	Male	12/06/2018	TMMC
CSL-14138	*Zalophus californianus*	Infected	No	Yes	Yes	Subadult	Male	12/23/2018	TMMC
CSL-B	*Zalophus californianus*	No known infection*	No	No	Yes	Adult	Female	Born in captivity	LIA
CSL-J	*Zalophus californianus*	No known infection*	No	No	Yes	Adult	Male	Born in captivity	LIA
CSL-S	*Zalophus californianus*	No known infection*	No	Yes	No	Adult	Female	Born in rehabilitation facility	NEAQ
CSL-T	*Zalophus californianus*	No known infection*	No	Yes	No	Adult	Female	In captivity since 2013	NEAQ

### 2.2 Parasite, fecal, and sputum sample DNA extractions

Total genomic DNA from whole nematode samples were isolated following established protocols, using organic phenol-chloroform extraction and ethanol precipitation (Keroack et al., 2016). DNA from fecal samples was isolated using the Qiagen DNeasy PowerSoil Kit (Hilden, Germany) following the manufacturer's protocols using a 0.5 g sample of feces. DNA was extracted from sputum (collected with nasal swabs) using the Qiagen DNeasy Blood & Tissue Kit (Hilden, Germany) following the manufacturer's protocols using a 0.5 g sample of sputum.

Purity of the DNA was checked using a Thermo Fisher Scientific nanodrop spectrophotometer (Waltham, MA, USA) and concentration was determined using a Thermo Fisher Scientific Qubit® 2.0 Fluorometer (Waltham, MA, USA) using 2 μL of the DNA sample with the Invitrogen dsDNA HS Assay Kit (Waltham, MA, USA).

### 2.3 Parasite identity confirmation by molecular barcoding

The internal transcribed spacer region 2 (ITS2) of whole nematode isolates was Sanger sequenced to confirm morphological species identifications (Supplementary Table 1). ITS2 was amplified using previously published nematode-specific primers, with 5′- AGTGCGAATTGCAGACGCATTGAG-3′ as the forward primer and 5′- AGCGGGTAATCACGACTGAGTTGA-3′ as the reverse primer (Rishniw et al., 2006). Amplification was done using Thermo Fisher Scientific's Phusion High-Fidelity PCR Kit (Waltham, MA, USA) under the following conditions: 98 °C for 3 min as an initial denaturing step; followed by 35 cycles of 98 °C for 30 s for denaturing, 60 °C for 30 s for annealing, and 72 °C for 1 min for extension; and a final extension of 72 °C for 10 min. PCR products were Sanger sequenced using Thermo Fisher Scientific's BigDye™ Terminator v3.1 Cycle Sequencing Kit (Waltham, MA, USA) following the manufacturer's protocol. Sequences were compared to the GenBank database using NCBI's BLASTn to confirm the species identity of the nematode samples (Altschul et al., 1990; Sayers et al., 2019).

### 2.4 Low-coverage genome sequencing (illumina)

Four *P. decorus* samples were pooled for low-coverage genome sequencing from hosts GFS-151, CSL-13271, CSL-13295, and CSL-13301 (Table 1). Genomic libraries for next-generation sequencing on Illumina's MiSeq System (San Diego, CA, USA) were fragmented using NEBNext® dsDNA Fragmentase® (Ipswich, MA, USA) following the manufacturer's protocol. The fragmentase reaction was incubated for 19 min to achieve a fragment profile averaging 550 base pairs. The sample size profile was confirmed using Agilent Technologies' Agilent 2100 Bioanalyzer with the Agilent Bioanalyzer High Sensitivity DNA Kit (Santa Clara, CA, USA) before continuing with the library preparation to ensure the desired fragment size was obtained.

Fragmented total genomic DNA was then modified using the NEBNext® DNA Library Prep Master Mix Set for Illumina® (San Diego, CA, USA) according to manufacturer's protocols with NEBNext® Multiplex Oligos for Illumina® (Ipswich, MA, USA) for indexing. Total input DNA for library construction was 540 ng. Library quality was validated using the Agilent Bioanlyzer High Sensitivity DNA kit (Santa Clara, CA, USA) and was sequenced using MiSeq Reagent Kit V3 and a 1% PhiX control (San Diego, CA, USA). The resulting sequences were converted into FASTQ files and had their adaptor and index sequences removed using BaseSpace® (basespace.illumina.com). Geneious 9.1.8 (https://www.geneious.com) was used to convert the sequences from FASTQ to FASTA files and to merge the paired reads using the FLASH plugin (Fast Length Adjustment of SHort reads, version 1.2.9, Magoc and Salzberg, 2011). The resulting sequence data can be found on NCBI's Sequence Read Archive (SRR11068184).

### 2.5 Repeat cluster generation

*P. decorus* genomic repeat family selection was done following an established, previously published workflow using standard parameters for all tools (Grant et al., 2019; Pilotte et al., 2019; Keroack et al., 2018; Papaiakovou et al., 2017; Pilotte et al., 2016; Nová;k et al., 2010, 2013). Unlike the workflows used in previous studies that only used sequences from one species to generate genomic repeat clusters, sequences from three outgroup species were included so that repeat family homology would be identified across the species, and repeat families present in other closely related species could be avoided in designing the diagnostic assay to ensure species-specificity. The closest species included in the sequencing was another pinniped-infecting lungworm in the metastrongylid group, *Otostrongylus circumlitus* (Chilton et al., 2006). Additionally, a non-metastrongylid strongylid was included, *Necator americanus*, as well as a non-strongylid nematode as the furthest outgroup, *Acanthocheilonema spirocauda* (Chilton et al., 2006). The samples included for the repeat cluster analysis and their sources are described in Supplementary Table 2. No low-coverage genomic data from a *Parafilaroides* species that was not *P. decorus* were available to use as an outgroup within the same genus.

Raw FASTA files from each species were uploaded to the Galaxy RepeatExplorer server (http://www. repeatexplorer.org/) (Nová;k et al., 2013). Once the FASTA files were uploaded to RepeatExplorer, files larger than one million reads were randomly subsampled down to one million reads to save computing time. Once subsampled as needed, the samples were head-to-tail concatenated to create one FASTA file containing sequences from *P. decorus* and the three outgroup species. Genomic repeat families were then identified using the RepeatExplorer Clustering tool on the concatenated FASTA sequences. The default settings were retained except the cluster size threshold for detailed analysis was lowered from 0.01% to 0.001% to maximize output.

### 2.6 Selecting a *P. decorus* repeat DNA family for qPCR assay design

**Fig. 1:**
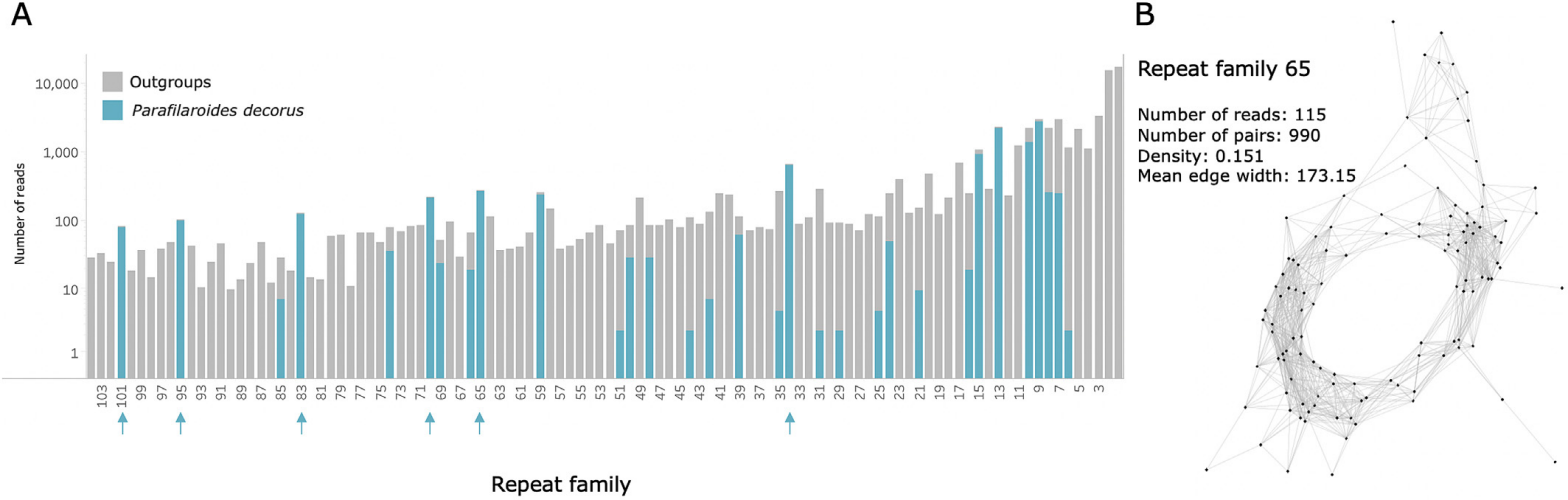
Repeat family selection for the *P. decorus* diagnostic assay. A. Number of sequencing reads for *P. decorus* compared to outgroup species reads for each repeat family (1–104) on a log scale. Arrows indicate repeat families with no reads from the outgroup species. Plot was made using Tableau Software, 2019. B. Within a cluster, reads with similar sequences are closer together. Edges connect a read with its closest match (creating a pair) and the length of this edge represents the amount of overlap between the reads. The mean edge width provides context for the lengths in the cluster, so in a cluster with a larger mean edge width the edges are actually longer than edges in a cluster with a smaller mean edge width. Reads therefore may be distant because of sequence divergence, or in the case of a long repeat (more than 150 base pairs), because of a lack of overlap between reads. However, because there will likely be continuous reads covering different regions of the repeat, these longer repeats should still appear as a tight, though possibly larger, cluster. Read dots that stray from the central cluster most likely represent sequence divergence. Higher density therefore indicates lower sequence divergence.

For each of the 104 repeat families discovered, the number of sequencing reads from *P. decorus* and each of the three outgroups that matched the repeat family were tallied. The number of reads from each species belonging to each of the repeat families is summarized in Fig. 1. For the diagnostic assay, a repeat family with zero reads in the three outgroups were selected as candidates. These candidates were then analyzed using BLASTn (http://blast.ncbi.nlm.nih. gov/Blast.cgi) to ensure that the candidate repeat families were not ribosomal or mitochondrial DNA sequences, or a close match to DNA sequences found in other related parasite species, the marine mammal host or human (in case of any contamination during DNA isolation and manipulation). Those repeat families showing any significant matches to other species were eliminated from consideration.

The RepeatExplorer Clustering tool uses the 3D version of the Fruchterman and Reingold algorithm to generate graph layouts of each repeat family, in which individual reads are represented by vertices and similar reads are connected by edges (Novák et al., 2010). Therefore, clusters composed of very similar reads will form tight clusters in the graph. To quantitatively measure the similarity of reads in a repeat family, the average edge length connecting reads is output for each repeat family. A smaller average edge length denotes greater similarity among reads. To maximize the percentage of the repeat family that would be captured by a single primer and probe set for a diagnostic assay, and therefore increase the sensitivity of the assay, only repeat families forming tight clusters in the graph layout with low average edge lengths were considered for the assay. For the purposes of this paper, a 2D projection of the selected repeat family graph layout is shown (Fig. 1B).

After removing those repeat families that had similarity to sequences in other species or did not form tight clusters, the repeat family with the most reads, and therefore the most abundant in the *P. decorus* genome, was selected for primer and probe design for a quantitative PCR assay. The repeat family selected was the second most abundant in the *P. decorus* genome of the original candidates. The cluster visualization of this repeat family and its associated statistics are presented in Fig. 1. For ease of discussion, this cluster was named Pd65 and the repeat family will henceforth be referred to as the Pd65 repeat. The full consensus sequence of the repeat family (GenBank accession no. MT053285) can be found in the supplementary material (Supplementary Sequence 1).

A primer-probe set (based on repeat family Pd65 identified in Fig. 1) for amplifying and detecting *P. decorus* DNA by qPCR was designed with the PrimerQuest tool offered through Integrated DNA Technologies (Coralville, IA, USA) using standard parameters (http://www.idtdna.com/primerquest/home/ index). The species-specificity of the primer-probe set was assessed using NCBI's Primer Blast tool (http://www.ncbi.nlm.nih.gov/tools/primer-blast/). The probe was labeled with a 6FAM fluorophore at the 5′ end and was double quenched using the internal quencher ZEN and the 3′ quencher 3IABkFQ (IOWA BLACK). This labeling/quenching combination has been shown to provide superior sensitivity to other probe/quencher systems (Pilotte et al., 2016).

The *P. decorus* repeat family Pd65 (Fig. 1) was amplified using 5′- GCA GAT AGG AAG AAC CCA CAA-3′ as the forward primer, 5′- AGC AAG CTG CTA ACC CTT C -3′ as the reverse primer, and/56-FAM/AC AGC AGT C/ZEN/A TCG TGT CCA TAC CA/3IABkFQ/ as the probe. The reactions were prepared using Thermo Fisher Scientific's TaqMan® Universal PCR Master Mix (Waltham, MA, USA) with 200 nM forward and reverse primer concentrations, and 125 nM probe concentration, following the manufacturer's protocols. Cycling conditions were as prescribed by the manufacturer with an annealing and extending temperature of 60 °C. One nanogram of input DNA was used for each reaction for a total reaction volume of 7 μL, a volume and concentration that has been proven optimal (Pilotte et al., 2016). All reactions were run on Thermo Fisher Scientific's StepOne Plus Real-Time PCR system (Waltham, MA, USA). All samples were run in quadruplicate. Mean C_q_ (quantification cycle) and standard deviation reported in the results were calculated using all four data points from each sample to reduce error.

### 2.7 Assay specificity and sensitivity testing

To test the species-specificity of the Pd65 primer-probe set, DNA from a variety of nematode species, including both metastrongylid and non-metastrongylid parasites, was qPCR amplified using the same conditions described above. DNA isolated from a confirmed *P. decorus* nematode from marine mammal host CSL-13308 was used as the positive control (Table 1). Whole nematodes used as negative controls were obtained from the following sources: Baylor College of Medicine (*Anclyostoma duodenale* and *Necator americanus*), Filariasis Research Reagent Resource Center (*Brugia malayi*), The Marine Mammal Center (*Otostrongylus circumlitus*), Mystic Aquarium (*Pseudoterranova decipens*), and National Marine Life Center (*Acanthocheilonema spirocauda* and *Contracaecum osculatum*). To determine the limits of detection for the assay, reactions were run with total input DNA of: 1 ng, 0.1 ng, 0.01 ng, 0.001 ng (1 pg), 0.0001 ng, 0.00001 ng, and 0.000001 ng (1 fg). Detection was reliable at 0.001 ng (1 pg) and had fairly low standard deviations; thus, this concentration was used for both specificity testing and testing of whole worm DNA isolates.

### 2.8 Testing of fecal and sputum samples

Fecal and sputum samples from otariids with known *P. decorus* infections and without known infections were provided by The Marine Mammal Center (Table 1). Fecal samples from one of the primary host species, *Zalophus californianus*, from the New England Aquarium were used as negative fecal controls because these animals have been in captivity for many years on a diet free of nematode-infected fish and were known to be free of *P. decorus* infections (Table 1). Similarly, sputum samples from *Zalophus californianus* were provided by the Long Island Aquarium for use as a negative control, as these sea lions had been born in captivity, fed on uninfected fish throughout their life, and were known to be free of *P. decorus* infections (Table 1). DNA from fecal samples and sputum samples was extracted as previously described. Pd65 qPCR was performed using 1 ng of template DNA per reaction and the reaction conditions described above.

## 3 Results

### 3.1 Specificity

To test the Pd65 qPCR assay for specificity, an array of nematode species ranging in phylogenetic distance from *Parafilaroides decorus* were tested, including *Otostrongylus circumlitus* within the same superfamily (Metastrongyloidea), *Necator americanus* within the same order (Strongylida), *Acanthocheilonema spirocauda* and *Brugia malayi* in the same class (Secernentea), and several nematode species outside of the Secernentea class (*Anclyostoma duodenale, Contracaecum osculatum,* and *Pseudoterranova decipiens*). Critically, we tested species that infect the same hosts as *P. decorus* (such as *Contracaecum osculatum*) to ensure that the assay does not identify false positives from species that will be present in the material being tested from the host species. Amplification was detected only in the *P. decorus* positive control, suggesting that the assay has high specificity at least to the genus level (Table 2). Samples from other *Parafilaroides* species that were not *P. decorus* were not available, thus specificity testing within the genus was not possible.

**Table 2:** Pd65 qPCR assay specificity testing.

Species	Input DNA (ng)	Mean Cq	Standard deviation	Source
*Parafilaroides decorus*	1	17.56	0.07915	The Marine Mammal Center
*Otostrongylus circumlitus*	1	No detection	–	The Marine Mammal Center
*Necator americanus*	1	No detection	–	Baylor College of Medicine
*Acanthocheilonema spirocauda*	1	No detection	–	National Marine Life Center
*Brugia malayi*	1	No detection	–	Filariasis Research Reagent Resource Center
*Contracaecum osculatum*	1	No detection	–	National Marine Life Center
*Pseudoterranova decipiens*	1	No detection	–	Mystic Aquarium
*Anclyostoma duodenale*	1	No detection	–	Baylor College of Medicine

### 3.2 Sensitivity

The sensitivity of the Pd65 qPCR assay for *P. decorus* was determined by testing pure *P. decorus* genomic DNA isolated from whole parasites in 1:10 serial dilutions ranging from 1 ng to 1 fg. Two positive control individual worm samples were used. Detection was possible in both samples down to 10 fg of DNA. However, the lower concentrations resulted in somewhat higher standard deviations of C_q_ values across replicates (Table 3). The lowest concentration that can be detected with confidence is 10 fg (or 0.00001 ng).

**Table 3:** qPCR assay sensitivity testing.

Input DNA (ng)	Host ID	Mean Cq	Cq standard deviation	Species
1	CSL-13301	16.98	0.09	*Parafilaroides decorus*
	CSL-13308	16.86	0.82	*Parafilaroides decorus*
0.1	CSL-13301	20.21	0.23	*Parafilaroides decorus*
	CSL-13308	18.74	0.88	*Parafilaroides decorus*
0.01	CSL-13301	23.75	0.04	*Parafilaroides decorus*
	CSL-13308	20.15	3.61	*Parafilaroides decorus*
0.001	CSL-13301	26.72	0.3	*Parafilaroides decorus*
	CSL-13308	25.8	1.23	*Parafilaroides decorus*
0.0001	CSL-13301	30.22	0.5	*Parafilaroides decorus*
	CSL-13308	29.07	1.28	*Parafilaroides decorus*
1E-05	CSL-13301	33.49	0.93	*Parafilaroides decorus*
	CSL-13308	33.07	1.33	*Parafilaroides decorus*
1E-06	CSL-13301	No detection	–	*Parafilaroides decorus*
	CSL-13308	No detection	–	*Parafilaroides decorus*

**Fig. 2:**
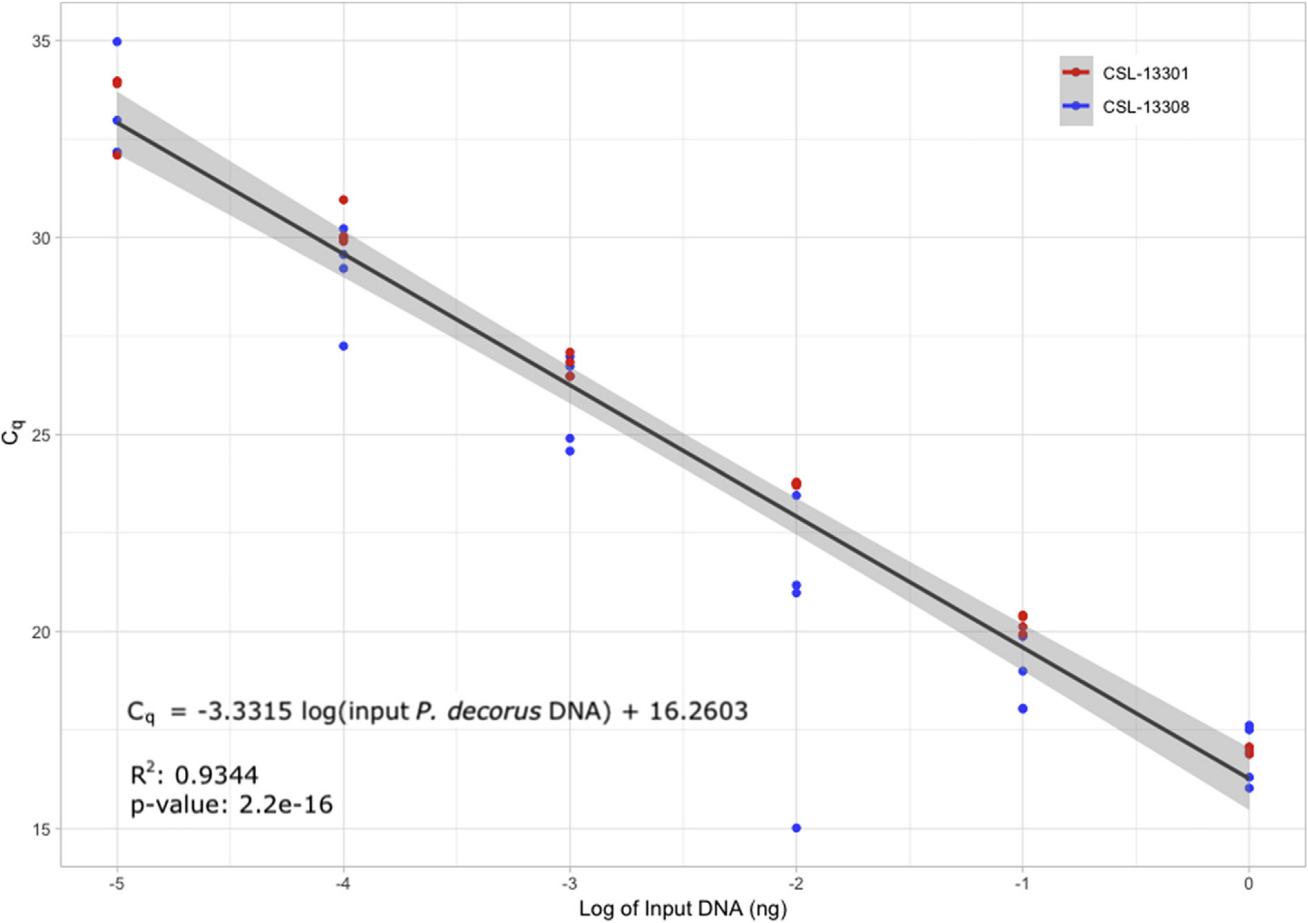
Standard curve based on sensitivity data. A 95% confidence interval for the linear regression model is shaded in grey.

The serial dilutions of the two positive control *P. decorus* DNA samples (CSL-13308 and CSL-13301) were used to develop a standard curve to estimate the concentration of *P. decorus* DNA in unknown samples, particularly in sputum and fecal samples (Fig. 2). The R^2^ value suggests that 93.44% of the variation in C_q_ value can be explained by the log of the amount of input target DNA.

### 3.3 Fecal samples from necropsied animals

C_q_ values obtained from the Pd65 qPCR on genomic DNA isolated from fecal samples containing unknown quantities of *P. decorus* were used to estimate the amount of *P. decorus* DNA that was in the sample. The amount of *P. decorus* DNA present in 1 ng of total fecal DNA (which would also include host DNA, bacterial DNA, etc.) is estimated in Table 4.

**Table 4:** Pd65 qPCR on DNA isolated from fecal samples. The amount of input *P. decorus* DNA was estimated using the equation in Fig. 2. Infection status was determined by fecal egg float or morphological identification of the worm discovered during necropsy. *Negative fecal controls: DNA isolated from the stool of animals living in aquaria that originated from outside of the parasites’ range and fed “sushi-grade” fish of species not known to carry *P. decorus.*

*P. decorus infection status*	Mean Cq	Cq standard deviation	Estimated input P. decorus DNA (ng)	Host ID
Infected	18.17	1.2	0.27	GFS-151
	25.41	0.024	0.0018	CSL-13302
	22.93	0.065	0.01	CSL-13541
	17.32	0.048	0.48	CSL-13564
	25.17	0.036	0.0021	CSL-13591
	36.33	0.91	9.40E-07	CSL-14040
	24.60	0.12	0.0031	CSL-14066
	24.88	0.57	0.0026	CSL-14070
	18.13	0.14	0.27	CSL-14073
	23.86	0.28	0.0052	CSL-14075
	33.80	0.093	5.40E-06	CSL-14083
	32.82	2.9	1.10E-05	CSL-14084
	17.15	0.35	0.54	CSL-14089
	19.37	0.57	0.12	CSL-14107
	28.71	0.17	0.00018	CSL-14117
	25.31	0.64	0.0019	CSL-14121
	20.09	0.60	0.071	CSL-14138
No known infection	No detection	-	–	CSL-13305
	25.47	0.084	0.0017	CSL-13534
No known infection*	No detection	-	–	CSL-S
	No detection	-	–	CSL-T

### 3.4 Paired fecal and sputum samples

C_q_ values obtained from Pd65 qPCR on genomic DNA isolated from paired fecal and sputum samples containing unknown quantities of *P. decorus* DNA were used to estimate the amount of *P. decorus* DNA that was in each sample. The amount of *P. decorus* DNA present in the 1 ng of total fecal and sputum DNA used in the Pd65 qPCR assay is estimated with a 95% confidence interval of ±0.39 ng in Table 5.

**Table 5:** Pd65 qPCR on genomic DNA isolated from paired fecal and sputum samples. Estimated amount of input *P. decorus* DNA is found using the equation in Fig. 2. Sputum samples from CSL-B and CSL-J were used as negative controls for sputum (Table 1). Both of these animals were born in captivity and fed “sushi-grade” fish of species not known to carry *P. decorus*. CSL-S and CSL-T fecal samples were used as negative controls as well (Table 1, Table 4). In all four of these samples, there was no detection of *P. decorus* DNA.

*Host ID*	Feces mean Cq	Sputum mean Cq	Feces estimated input target DNA (ng)	Sputum estimated input target DNA (ng)
CSL-14040	36.33	26.08	9.45E-07	0.001132
CSL-14066	24.6	16.55	0.00314	0.8204
CSL-14070	24.88	31.83	0.00258	2.12E-05
CSL-14073	18.13	20.63	0.2741	0.04866
CSL-14075	23.86	28.11	0.005244	0.0002778
CSL-14083	33.8	31.03	5.42E-06	3.68E-05
CSL-14084	32.82	24.65	1.07E-05	0.003039
CSL-14089	17.15	29.69	0.5406	9.33E-05
CSL-14107	19.37	18.7	0.1166	0.1858
CSL-14117	28.71	28.26	0.0001832	0.0002497
CSL-14121	25.31	32.26	0.001925	1.58E-05
CSL-14138	20.09	26.03	0.07093	0.00117

*P. decorus* detected in both sample types and there was never detection in negative fecal controls or negative sputum controls. Neither sample type consistently produces higher DNA concentration estimates than the other.

## 4 Discussion

### 4.1 Specificity

The qPCR assay developed here appears to be genus specific to *Parafilaroides*, as no other nematode species were detected by the assay. We cannot say definitively that the assay is species-specific to *P. decorus* because no nematode species of the same genus (*Parafilaroides*) were tested. Due to the extremely small size of the parasite and their tendency to be embedded in the pulmonary tissue, most facilities do not collect *Parafilaroides* specimens during necropsy. However, no other *Parafilaroides* species are known to infect the pinnipeds *P. decorus* classically infects (the California sea lion, Guadalupe fur seal, and Northern fur seal) (Kuzmina et al., 2018). Whether our assay would detect these other species is unknown. While *Parafilaroides* is not routinely collected during most necropsies, we hope to obtain *Parafilaroides* samples of other species in the future to determine if the assay is species-specific, or if it amplifies DNA from other *Parafilaroides* species as well. Despite these unknowns, the level of specificity of the Pd65 assay still surpasses the barcoding PCR tests currently in use (Rishniw et al., 2006).

### 4.2 Sensitivity

In pure *P. decorus* genomic DNA samples, detection of as little as 10 fg, or 0.00001 ng, was possible (Table 3). To discern the relationship between DNA concentration and number of larvae, an exact larval count is needed in biological samples. While such data do not exist for *P. decorus*, a previous *Ascaris lumbricoides* study have found roughly 1000 eggs yielded 1 ng of nematode DNA from fecal samples (Easton et al., 2017). This would suggest that our qPCR assay can detect the presence of 0.01 eggs, assuming *A. lumbricoides* and *P. decorus* have similar quantities of DNA per egg or larva respectively, and assuming efficient isolation of DNA from biological material.

### 4.3 Detection of *P. decorus* in fecal samples

Using the Pd65 qPCR assay, *P. decorus* was detected in fecal samples from all animals with known *P. decorus* infections. Furthermore, all of the estimates of the amount of *P. decorus* DNA found in the fecal samples were less than the total 1 ng of DNA in the fecal sample (at a maximum of 48% of the total DNA), which suggests that the *P. decorus* DNA estimates are likely correct, since most of the DNA in a fecal sample should be host and bacterial DNA. Unfortunately, we cannot directly estimate the concentration of larvae in the feces because the correlation between egg DNA concentration in the feces and number of larvae is currently unknown for *P. decorus*.

Host CSL-13534, notably, was not known to have a *P. decorus* infection based on the fecal testing and necropsy data (Table 1, Table 4). Infections with these particularly small nematodes can be easily missed in necropsies and, more importantly, in fecal Baermann tests that can be processed while the animal is still alive. This demonstrates that the Pd65 assay can detect infections that would otherwise be missed. This scenario is similar to soil transmitted helminth molecular diagnostic assays, which are often able to detect infections that are missed by visual fecal examination methods due to storage methods causing eggs to break down or human error (Pilotte et al., 2016, 2019; Easton et al., 2017).

### 4.4 Detection of *P. decorus* in paired fecal and sputum samples

*P. decorus* detection was achieved by the Pd65 qPCR assay in both fecal and sputum samples from all hosts tested with known *P. decorus* infections. Furthermore, both feces and sputum samples were equally sensitive for detection of *P. decorus* DNA (Table 5). This suggests that either or both of these sample types could be used to diagnose *P. decorus* infection using this assay. However, feces and sputum were not necessarily collected on the same day, and therefore may have been collected during different points in the course of the infection, or even during different points in treatment of the infection. Thus, the true relationship between Pd65 qPCR detection in feces and sputum as indicators of *P. decorus* infection warrants further investigation. A follow-up study to examine this relationship using paired fecal and sputum samples collected at the same time points is necessary.

## 5 Conclusions

The Pd65 repeat-based qPCR assay will enable the marine mammal rehabilitation community to use fecal or sputum samples to diagnose and treat live animals with confidence in the identity of *P. decorus* infections and with far greater specificity and sensitivity than is possible with fecal Baermann tests or other microscopy-based methods. Even with the limited number of samples, the Pd65 qPCR assay has already uncovered an undetected *P. decorus* infection in a sea lion that had previously gone undiagnosed.

The Pd65 qPCR assay also has the potential for use in screening wild populations for infection using scat to obtain data on the epidemiology of infection under natural conditions. As demonstrated here, the assay is able to identify a *P. decorus* infection and the comparative level of burden in live animals using either feces or sputum. This information can be used in the future to best determine the optimal treatment to aid in the rehabilitation and release of infected animals.

## Declaration of competing interest

None.
